# Influence of child structure and means of eldercare on equitable distribution of family assets

**DOI:** 10.1371/journal.pone.0312921

**Published:** 2024-11-14

**Authors:** Yangyang Chen, Hao Ji

**Affiliations:** 1 College of Economics and Management, Zhejiang A&F University, Hangzhou, People’s Republic of China; 2 Center for Medical Intelligence and Health Policy Research, Hangzhou Medical College, Hangzhou, People’s Republic of China; Indian Institute of Technology Jodhpur, INDIA

## Abstract

To provide a reference for the realization of children’s equal inheritance, this study examined the influence of child structure and means of eldercare on equitable family property distribution willingness. Using data from surveys of Chinese women’s social status, this study analyzes the impacts of child structure and means of eldercare on family property distribution, with the children’s surname choice and women’s status as moderating factors. The results indicate that a child structure in which the family consists of more girls than boys, a higher number of children, and the choice of family support for eldercare significantly inhibit the equal distribution of family assets. However, the weakening of traditional views significantly promotes the equal distribution of household assets. These findings remain robust after endogenous and robustness tests. Moreover, the study finds that the impact of the structure of children and eldercare methods varied across different age groups, household registration attributes, and genders. Research on the willingness to distribute household wealth aids in understanding the effects and differences brought about by the structure of children and eldercare methods under current traditional cultural contexts, thereby promoting a more equitable distribution of household wealth.

## Introduction

Throughout history, societal development has continued to challenge the belief that heroes are pre-determined according to one’s birth circumstances. However, during China’s period of rapid economic growth, the emergence of significant wealth disparity led to the division between the rich and poor second generations. This trend implies that intergenerational wealth transfer plays a crucial role in enhancing future generations’ welfare and is an essential avenue for wealth accumulation of descendants. However, the unequal distribution of family property has led to an imbalanced development of wealth accumulation among the descendants of the family [1]. Household wealth is a focal point in economic and sociological research, with the distribution of household wealth being a core issue in household behavioral economics. Investigating the distribution of household wealth is crucial for promoting the equitable transfer of assets among generations. For children, the direct allocation of household wealth leads to rapid asset accumulation, enhancing their quality of life. Alternatively, household wealth indirectly benefits children by funding their education, thereby increasing their chances of obtaining higher-paying jobs and achieving a better standard of living [2–4]. From the parents’ perspective, distributing wealth to their children is often motivated by the desire to receive more support and care in their old age [5]. Traditional Chinese society, heavily influenced by Confucianism, has integrated gender norms into the process of intergenerational wealth transfer [6, 7], developing a family property relationship system where wealth is passed down from father to son [8]. This system is deeply rooted in the conflict between power and obligation and the clash between customs and laws.

The decline of traditional practices and the rise in women’s economic status marked by modernization have weakened traditional family values, including those related to property inheritance and redistribution [9]. However, despite children’s ability to provide financial support [10], a corresponding alignment between the power of property inheritance and the obligation of financial support has not emerged [11]. The changes observed in the equitable property distribution willingness may be due to the constraints imposed by population policies [12]. In this context, the contradiction between parents’ decision-making process and children’s bargaining mechanism have increased conflicts on family property distribution and children’s financial responsibilities to their parents [13].

The distribution of family wealth is often influenced by various factors such as the family’s economic conditions [14–16], demographic structure [17, 18], number of children [19–21], and family members’ health statuses [22]. Some scholars have examined elderly people’s preference for male inheritance and theoretically divided the economic relationships between parents and children based on the downward transfer of wealth [23, 24]. However, the intentions underlying family property distribution and the internal dynamics related to child structure and eldercare arrangements within the family have received limited attention. Considering different age groups, household attributes, gender structures, and other factors, this study examined the influence of child structure and eldercare arrangements on family property distribution to provide substantial data and empirical findings for the realization of equitable inheritance among children.

## Materials and methods

### Data sources

Data were obtained from the second and third phases of the China Women’s Social Status Survey, comprising information on demographics, employment, income, and political participation. The sampled data, which includes 31 Chinese provinces (including municipalities and autonomous regions), has been made available to the public in the years 1990, 2000, and 2010. Data from 1990 are not able to provide sufficient information on child structure and used a smaller sample size. Nevertheless, data from 2000 and 2010 include records of families’ property distribution intentions from parents’ perspectives and comprehensive research on child structure and eldercare choices, thus providing more detailed information pertinent to this study. To meet the research objectives, we integrated data from 2000 and 2010 as robust support to explore the potential impact of differences in child structure and eldercare choices on intergenerational property transfer within families. Additionally, to avoid potential biases caused by property distribution intentions of families with single-gender children, samples without children and with single-gender children were excluded. After excluding data with missing values, the final sample comprised 10,689 cases. The data were derived from cross-sectional national statistics, employing robust survey methodologies, providing estimates that were both nationally representative and internationally comparable [25]. The resulting estimates were nationally representative and internationally comparable.

### Data analysis

The dependent variable was employed through the following question: “What is your opinion on how a married daughter should inherit family property?” Respondents who endorsed the equal division of property among married daughters and their brothers were designated as inclined toward equal distribution and assigned a value of 1. Conversely, those who expressed contrasting views, such as married daughters should inherit less or more than their brothers or should not inherit family property, were categorized as disinclined toward equal distribution and assigned a value of 0. Furthermore, within the subset of respondents who exhibited reluctance toward equal distribution, we conducted the following analysis. Those who indicated that married daughters should inherit less or more than their brothers were classified as amenable to allocating family property to daughters and assigned a value of 1. Those who indicated that married daughters should not inherit family property were classified as disinclined to allocate family property to daughters and assigned a value of 0. Respondents who provided an unclear response were systematically excluded from the dataset owing to definitional challenges and their limited representation.

The key explanatory variables comprise both child structure and approach to eldercare. Child structure includes children’s gender structure and the number of children in the family. Gender structure and the number of children were discrete and continuous variables, respectively. To categorize eldercare approaches, respondents were asked “How do you plan to support yourself in old age?” Those who indicated a preference for relying on family support were assigned a value of 1 and those who indicated a preference for relying on commercial insurance, a retirement pension, a pension fund, and social assistance were assigned a value of 0. Notably, the 2000 survey did not directly ask this question; however, we treated the response “No retirement pension, no old age insurance, no property or savings but can rely on family care or financial support” as equivalent to a respondent’s intention to rely on family support, which was assigned a value of 1, whereas all other responses were assigned a value of 0.

The control variables were the following basic demographic variables: household registration (*hukou*), gender, birth cohort, education level, marital status, and health status. The economic capacity variables included whether the respondent had social old age security and whether they were engaged in income-generating labor. Family status characteristics referred to respondents’ willingness to allow children to take their mother’s surname and their answer on which parent holds more power within the family. Considering the significant differences in the number of children born during the implementation of China’s one-child policy in 1979 and a potential sample distribution biases, we incorporated the implementation of the 1979 one-child policy as a categorical variable, with the following categories: before 1960, 1960–1979, and after 1979. An overview of the basic information on the study variables is provided in Table 1.

**Table 1:** Sample characteristics.

Variable	Category and units	Mean	SD
Family property distribution intention			
Whether equal distribution exists in the family	1 = Yes,0 = No	0.454	0.498
Whether to distribute property to the daughter in an unevenly distributed family	1 = Yes,0 = No	0.399	0.490
Child structure			
Gender structure	1 = Daughters get more than sons,0 = Otherwise	0.221	0.415
Number of children	The unit	2.675	0.941
Daughter ratio	%	0.510	0.118
Method of eldercare	1 = Family care,0 = Otherwise	0.357	0.479
Control variable			
Household registration	1 = Agricultural households,0 = Non-agricultural households	0.751	0.433
Gender	1 = Female,0 = Male	0.532	0.499
Birth cohort			
Before 1960	1 = Yes,0 = No	0.580	0.494
1960 to 1979	1 = Yes,0 = No	0.409	0.492
After 1979	1 = Yes,0 = No	0.011	0.103
Education	0 = Illiterate or barely literate,6 = Primary school,9 = Middle school,12 = High school/Technical secondary school,15 = Junior college,16 = Undergraduate course,19 = Master	7.422	3.628
Marital status	1 = Married and with a spouse,0 = Divorce/Widowhood	0.999	0.031
Health status	1 = Very good or Good,0 = Average, Poor, or Very poor	0.900	0.300
Whether there is a social pension security?	1 = Yes,0 = No	0.312	0.463
Whether they are engaged in gainful labor?	1 = Yes,0 = No	0.816	0.388
Would you like your child to take her mother’s last name?	1 = Yes,0 = No/Indifferent	0.304	0.460
Who has more power in the family?	1 = Wife/Equality between spouses, 0 = Husband	0.554	0.497

### Models

First, we examined whether child structure and retirement arrangements impacted family property distribution. We further analyzed their influence on inheritance allocation to daughters in families with an unequal wealth distribution. Second, our research delved deeper into the effects of children’s gender structure on family property distribution intentions within the changing landscape of traditional values and women’s evolving status by considering the moderating roles of children’s surname selection and females’ status. Additionally, we conducted endogeneity and robustness tests. Finally, the sample was categorized by age group, household type, and gender, which served as reference groups for each other. This approach allowed for a comparison of the heterogeneity of the impacts of child structure and retirement arrangements on family property distribution intentions.

#### Econometric model

Factors including child structure and retirement arrangements were measured using the constructed Model (1) to comprehensively investigate the differences in family property distribution willingness.


$$Y_{i}=\alpha_{i}+\beta D_{i}+\gamma Z_{i}+\varepsilon_{i}$$ (1)

The explanatory variable *Y*_*i*_ reflects the willingness of *i* sample family to distribute property, which is expressed by “whether it is equally distributed” and “whether daughters are distributed in inequality.” *D*_*i*_ represents the factors of child structure and pension style of the *i* sample. *β* represents the marginal effect of the independent variable on the dependent variable given the other control variables. *α*_*i*_ is the household fixed effect, *z* is the control variable, time effect, and regional effect, and *ε*_*i*_ is the random interference term.

We employed the logit model to estimate binary variables, such as gender structure and eldercare methods, and used the OLS model to estimate continuous variables, such as the number of children. The advantages of the logit model and the OLS estimation lie in their foundation on a linear model framework, which allows for parameter estimation and the interpretation of relationships between variables. Both models facilitate model fitting, diagnostics, multivariable analysis, and hypothesis testing. They offer greater flexibility and accuracy than other models when dealing with cross-sectional data.

#### Moderating effect model

To further test the moderating effect of surname choice on gender structure, we constructed the following model:

$$Y_{i}=\alpha_{i}+\alpha_{1}XB_{i}+\alpha_{2}NAME_{i}+\alpha_{3}XB_{i}\times NAME_{i}+\alpha_{4}Z_{i}+\varepsilon_{i}$$ (2)

Where *XB*_*i*_ is the gender structure, *NAME*_*i*_ is the choice of surname, *XB*_*i*_×*NAME*_*i*_ is the interaction term between gender structure and surname choice, *α*_1_, *α*_2_, *α*_3_, and *α*_4_ are the coefficients to be estimated, and *α*_*i*_, *Z*, and *ε*_*i*_ are the household fixed effect; the control variable, time effect, and regional effect; and the random interference term, respectively.

#### Propensity score matching (PSM)

To effectively control sample selection bias, we used propensity score matching. This method relies on the assumption of conditional independence given common support and introduces the fundamental idea of a counterfactual framework. We constructed unobservable counterfactual outcomes to objectively compare the differences in the effects due to the child structure between the treatment and control groups.

#### Heterogeneity analysis

To consider individual differences, we conducted a heterogeneous analysis encompassing various life stages, household attributes, and gender structures. Given that family property distribution intentions are subject to the multifaceted influences of individual, familial, and societal factors, considerable disparities can be observed. Consequently, we conducted an in-depth examination of the heterogeneous effects of child structure and retirement mode on the inclination toward the equitable distribution of family wealth. The investigation was conducted according to the classification by age, various household factors, and gender distinctions.

## Results

### Influence of child structure and means of eldercare

Baseline regression estimates for the impacts of child structure and retirement arrangements on family property distribution intentions are reported in Table 2. Controlling for temporal and regional effects, Models (1)–(4) indicated a significant decrease in equal wealth distribution in families with a higher number of children, with more female children than male children, and with preference for family-based eldercare, all at the 1% confidence level. Models (5)–(8) further explored the influences of child structure, number of children, and retirement arrangements on the allocation of family wealth to daughters in families characterized by unequal wealth distribution. The results revealed that a higher number of children and family-based eldercare significantly inhibited the allocation of wealth to daughters at the 1% significance level. However, the findings of Models (6) and (8) regarding child structure diverged from those of Model (5). Despite the unfavorable effect of a higher number of children on wealth allocation to daughters, families with more female children than male children exhibited a tendency to allocate wealth to daughters as the number of children increased.

**Table 2:** Hierarchical regression of child structure, means of eldercare, and family property distribution intention.

Variable	Whether equal distribution			
	(1)	(2)	(3)	(4)
Gender structure	-0.358*** (0.051)	-0.181*** (0.058)	——	-0.187*** (0.058)
No. of children	——	-0.173*** (0.027)	——	-0.147*** (0.027)
Method of old-age care	——	——	-0.627*** (0.045)	-0.600*** (0.045)
Time effect	Control	Control	Control	Control
Regional effect	Control	Control	Control	Control
No. of samples	10689	10689	10689	10689
	Whether to distribute property to the daughter in an unevenly distributed family			
	(5)	(6)	(7)	(8)
Gender structure	-0.063 (0.074)	0.080 (0.082)	——	0.072 (0.083)
No. of children	——	-0.147*** (0.039)	——	-0.130*** (0.039)
Method of eldercare	——	——	-0.537*** (0.067)	-0.524*** (0.067)
Time effect	Control	Control	Control	Control
Regional effect	Control	Control	Control	Control
No. of samples	5827	5827	5827	5827

Based on the regression results, families with more girls than boys are likely to exhibit a “boy preference,” leading to unequal distribution of household wealth. Although an increase in the number of children generally hinders equal distribution, it also reduces the degree of inhibition caused by gender structure. In other words, while having more children does not change the state of unequal distribution, it can mitigate the concentration of household wealth caused by gender structure.

However, in families where gender discrimination already exists, the gender structure no longer plays a decisive role. As the number of children increases, not only does it fail to weaken the discriminatory allocation of assets, but it also intensifies the concentration of wealth towards boys. In such families, the likelihood of girls receiving a share of the property decreases as the number of children increases. A possible explanation is that families with more girls and a larger number of children are more likely to practice gender discrimination, making it difficult to achieve equal distribution of assets. Furthermore, family-based eldercare not only hinders the equal distribution of household wealth but also disadvantages daughters in the allocation of property. The traditional belief of “raising sons to support old age” continues to strongly influence the flow of household wealth.

### Effect of child structure

The regression results of the impact of child structure on family property distribution intentions, including the control variables, are reported in Table 3. After gradually adding the population, individual economic capacity, and family status variables, the results of the benchmark model displayed good robustness. Models (1)–(3) regressed the whole sample; the results revealed a significantly negative influence of child structure on the equal distribution of family property at the 1% level, with a higher number of female children than male children significantly inhibiting the equal distribution of property. The number of children significantly negatively impacted the equal distribution of family property at the 1% level, with a higher number of children inhibiting equal distribution of property. Models (4)–(6) were regressive samples of families with unequal wealth distribution. The results indicated that the child structure promoted the distribution of family property to daughters; families with more female children than male children allocate property to daughters. However, this finding failed the significance test. Moreover, a higher number of children had a significant negative effect on family property distribution to daughters; the more children in the family, the lower the inclination to allocate wealth to daughters. These results are consistent with the previous reference regression results, indicating the robustness of our results.

**Table 3:** Effect of child structure on family property distribution intention.

Variable	Whether equal distribution			Whether to distribute property to the daughter in an unevenly distributed family		
	(1)	(2)	(3)	(4)	(5)	(6)
Child structure						
Gender structure	-0.153*** (0.060)	-0.156*** (0.060)	-0.163*** (0.061)	0.079 (0.084)	0.077 (0.084)	0.079 (0.084)
No. of children	-0.078*** (0.029)	-0.077*** (0.029)	-0.070** (0.030)	-0.085** (0.042)	-0.087** (0.042)	-0.082* (0.042)
Control variable						
Domicile	-1.034*** (0.055)	-0.881*** (0.063)	-0.841*** (0.064)	-0.858*** (0.087)	-0.649*** (0.100)	-0.635*** (0.100)
Gender	0.051 (0.045)	0.048 (0.046)	-0.070 (0.047)	-0.133* (0.068)	-0.151** (0.069)	-0.216*** (0.070)
Birth cohort						
1960 to 1979	0.001 (0.048)	0.047 (0.049)	0.045 (0.050)	0.008 (0.071)	0.048 (0.072)	0.048 (0.072)
After 1979	0.391* (0.216)	0.437** (0.215)	0.457** (0.219)	-0.297 (0.365)	-0.265 (0.361)	-0.250 (0.356)
Education	0.084*** (0.007)	0.078*** (0.007)	0.072*** (0.007)	0.043*** (0.010)	0.039*** (0.010)	0.036*** (0.011)
Marital status	-0.505 (0.689)	-0.445 (0.673)	-0.430 (0.702)	1.100 (0.925)	1.147 (0.938)	1.177 (0.976)
Health status	0.013 (0.075)	0.021 (0.075)	0.026 (0.077)	0.396*** (0.115)	0.420*** (0.116)	0.445*** (0.117)
Whether there is social pension security?	——	0.326*** (0.053)	0.289*** (0.054)	——	0.315*** (0.085)	0.301*** (0.085)
Whether they are engaged in gainful labor?	——	-0.107 (0.066)	-0.128* (0.068)	——	-0.252** (0.103)	-0.263** (0.103)
Would you like your child to take her mother’s last name?	——	——	0.776*** (0.048)	——	——	0.431*** (0.078)
Who has more power in the family?	——	——	0.339*** (0.045)	——	——	0.133** (0.066)
Time effect	Control	Control	Control	Control	Control	Control
Regional effect	Control	Control	Control	Control	Control	Control
No. of samples	10689	10689	10689	5827	5827	5827

The results for the control variables generally align with expectations. Families with agricultural households are more unequal in wealth distribution than non-agricultural households and are less likely to allocate property to daughters. This may be due to the deeper influence of traditional ideologies and a stronger preference for sons in rural areas. Gender was not a significant factor in whether household wealth was distributed equally, but it had a significant negative impact on whether property was allocated to daughters. Females were less likely than males to allocate property to daughters.

The birth cohort variable indicated that younger families were more inclined towards equal distribution of wealth, but this did not significantly affect whether the property was allocated to daughters. In families where gender discrimination already existed, younger age did not improve the distribution of assets. As education levels increased, gender discrimination among children weakened, promoting more equitable wealth distribution and a greater tendency to allocate property to daughters.

Good health status did not significantly promote the equal distribution of household wealth but did significantly enhance the allocation of property to daughters. Healthier individuals are less dependent on family support, reducing the need for sons to provide eldercare, and are thus more inclined to allocate property to daughters. Individuals with social pension security were more likely to distribute wealth equally and allocate property to daughters than those without. This may be because individuals with stronger personal retirement capabilities rely less on sons for eldercare, reducing the likelihood of gender-biased wealth distribution.

It was found that engaging in gainful work hindered both the equal distribution of wealth and the allocation of property to daughters. This could be because a smaller proportion of income is saved, leading to less accumulated wealth that can only support single-lineage inheritance. Families willing to let their children take the mother’s surname were likely less influenced by traditional succession ideologies, promoting fair wealth distribution and aiding in allocating property to daughters. Finally, when women held more real power within the family, it facilitated both equal wealth distribution and the allocation of property to daughters.

### Influence of retirement mode

Building upon Table 3, the results of incorporating additional variables related to retirement arrangements and investigating the relationship between retirement mode and family property distribution intentions are reported in Table 4. Considering the potential correlations and heterogeneity among number of children, child structure, and retirement arrangements, the regression was conducted in subgroups. The results revealed that while controlling for other variables, retirement mode consistently exhibited a significant negative effect on family property distribution intentions at the 1% confidence level. Models (1) and (2) indicated that, compared to families with two children, having three or more significantly inhibited equal wealth distribution and the allocation of wealth to daughters, with the coefficients of those gradually increasing and decreasing, respectively. Models (3) and (5) demonstrated that a higher number of daughters, especially when daughters constituted the larger proportion of children in the family, hinders equal wealth distribution. Such families may favor sons over daughters. Furthermore, some families continue having children until a son is born, which leads to a higher proportion of daughters, concentrating the allocation of resources toward sons. Models (4) and (6) established that in families with unequal distribution of wealth, a higher number of daughters than sons did not significantly affect the distribution of wealth to daughters; however, as the number of daughters increased, the coefficient gradually shifted from negative to positive. This suggests that in families with pre-existing gender discrimination, the likelihood of daughters receiving inherited wealth decreased with an increase in the number of children, consistent with the previous findings.

**Table 4:** The influence of retirement mode on family property distribution intention.

Variable	Whether equal distribution exists in the family	Whether to distribute property to the daughter in an unevenly distributed family	Whether equal distribution exists in the family	Whether to distribute property to the daughter in an unevenly distributed family	Whether equal distribution exists in the family	Whether to distribute property to the daughter in an unevenly distributed family
	(1)	(2)	(3)	(4)	(5)	(6)
Method of eldercare	-0.278*** (0.051)	-0.314*** (0.074)	-0.283*** (0.051)	-0.317*** (0.074)	-0.287*** (0.051)	-0.317***(0.074)
No. of children (Reference class: 2)						
3	-0.195*** (0.052)	-0.176** (0.076)	——	——	——	——
≥4	-0.270*** (0.069)	-0.166* (0.099)	——	——	——	——
No. of daughters (Reference class: 1)						
2	——	——	-0.212*** (0.056)	-0.129 (0.080)	——	——
≥3	——	——	-0.232** (0.090)	0.063 (0.127)	——	——
Daughter ratio			——	——	-0.392** (0.185)	0.376 (0.262)
Control variable	Control	Control	Control	Control	Control	Control
Time effect	Control	Control	Control	Control	Control	Control
Regional effect	Control	Control	Control	Control	Control	Control
Number of samples	10689	5827	10689	5827	10689	5827

### Moderating effects of children’s surname choice and women’s status

Children can inherit either their father’s or mother’s surname, but historically, the practice of assigning the paternal surname has remained predominant. Hence, sons are endowed with the responsibility of surname continuity, a fundamental element in the intergenerational transfer of wealth based on the principle that only males can be heirs. Given the scarcity of data on open surname inheritance, to explore the evolution of gender norms in traditional culture, we employed willingness to assign children the maternal surname as a proxy variable to examine the surname selection tendency in families. Additionally, academic research on the advancement of women’s status and gender equality revealed that women’s status in China is gradually improving, and society is advancing toward gender equality [26]. Against this backdrop, the present study used the variable of which parent holds more power in the family to represent changes in women’s status. The moderating effect of the variable was examined, and the results are presented in Table 5.

**Table 5:** The moderating effect of children’s surname choice and women’s status.

Variable	Whether equal distribution exists in the family	Whether to distribute property to the daughter in an unevenly distributed family	Whether equal distribution exists in the family	Whether to distribute property to the daughter in an unevenly distributed family
	(1)	(2)	(3)	(4)
Gender structure	-0.308*** (0.066)	-0.054 (0.086)	-0.244*** (0.080)	0.140 (0.106)
Gender structure×Surname choice	0.252** (0.116)	0.270 (0.184)	——	——
Surname choice	0.720*** (0.054)	0.367*** (0.087)	——	——
Gender structure×Female status	——	——	0.029 (0.108)	-0.273* (0.152)
Female status	——	——	0.336*** (0.050)	0.205*** (0.075)
Control variable	Control	Control	Control	Control
Time effect	Control	Control	Control	Control
Regional effect	Control	Control	Control	Control
No. of samples	10689	5827	10689	5827

Models (1) and (2) estimated the impact of an interaction term between child structure and children’s surname selection on family property distribution intentions. The results indicated that the coefficients of the interaction term were both positive, with Model (1) being statistically significant at the 5% level. This suggests that surname selection positively moderated the effect of child structure on whether family wealth would be equally distributed, thereby effectively mitigating inequality owing to the child structure. Additionally, surname selection significantly promoted equitable family property distribution, but the interaction term with child structure was not significant in Model (2). This further indicates that the positive moderating effect of surname selection on family property distribution intentions primarily manifests in whether family wealth is equally distributed. The decline of traditional cultural practices promoted the equal distribution of family property but did not impacted inequality among families.

Models (3) and (4) estimated the moderating effect of women’s status. The results showed that the coefficient of the interaction term between child structure and women’s status was positive and negative and statistically significant at the 10% level in Models (3) and (4), respectively. This suggests that women’s status elevation did not significantly moderate family property distribution intentions but had a noticeable negative moderating effect on whether daughters were allocated wealth in families with unequal wealth distribution. This implies that the elevation of women’s status negatively moderated the distribution of wealth on daughters in families with pre-existing gender discrimination. This indicates that mothers may have experienced more severe gender discrimination in these households. In summary, the moderating effect of changes in women’s status on family property distribution intentions was not evident. This may be explained by the tradition of mothers passing wealth to sons in families with pre-existing gender discrimination.

### Pathway analysis of influences

**Fig 1:**
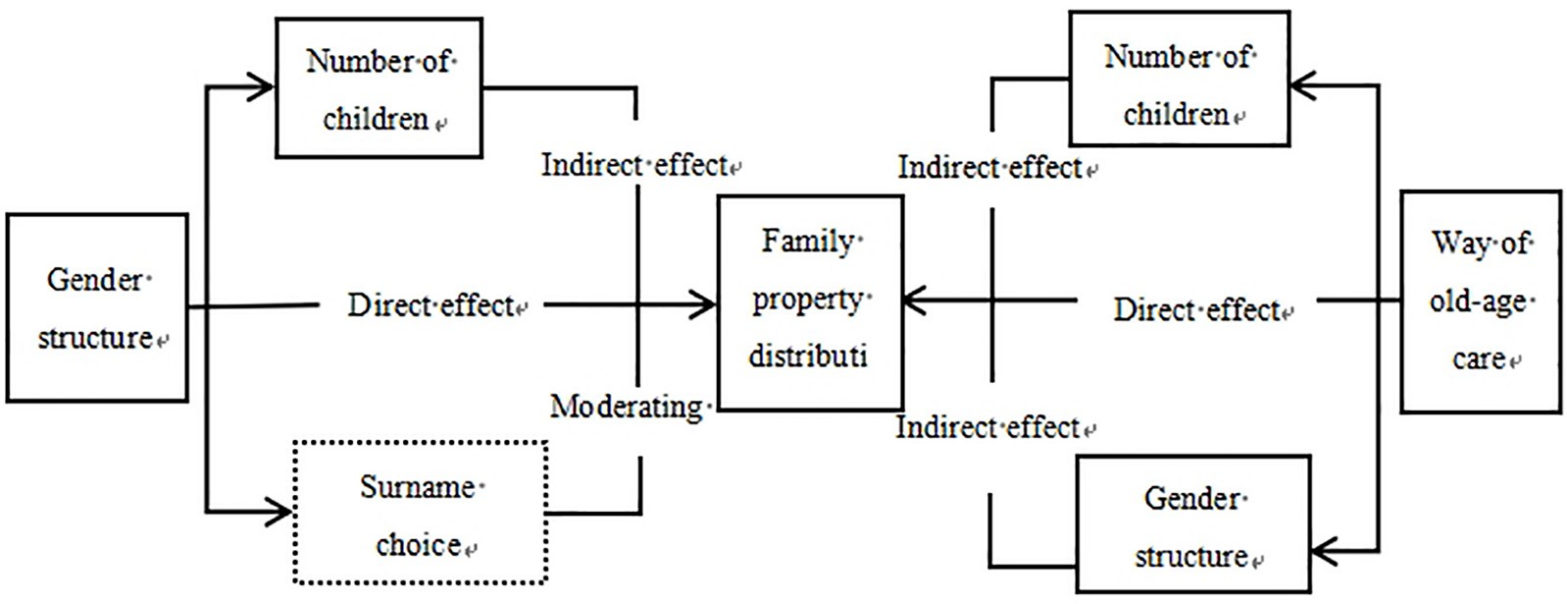
Conceptual framework.

Based on the research conclusions above, we constructed a pathway analysis diagram to illustrate the influences. Fig 1 illustrates a path analysis demonstrating the impacts of child structure, eldercare arrangements, and family property distribution intentions. The results show an indirect effect of child structure on family property distribution intentions induced by competition due to unequal gender distribution and the direct consequences of gender structure disparities. A higher number of children diminishes the unequal family property distribution based on gender. However, the impact of child structure should be analyzed considering both its inherent structure and the evolving traditional family values. This study employed surname selection as a moderating factor to explore the effects of the decline in traditional gender norms on family property distribution intentions.

In addition to the direct implications of various retirement modes, choosing family members to care for the elderly may increase childbirth rates, thereby influencing family property distribution intentions through the intergenerational caregiving decision-making mechanism. Conversely, influenced by the child structure, the practice of raising children for eldercare inclines family property distribution intentions toward sons [27, 28]. Parents’ solitary decision-making process also determines family property distribution intentions [29]. In summary, retirement arrangements can indirectly impact family property distribution intentions through these two avenues.

### Endogeneity and robustness tests

**Fig 2:**
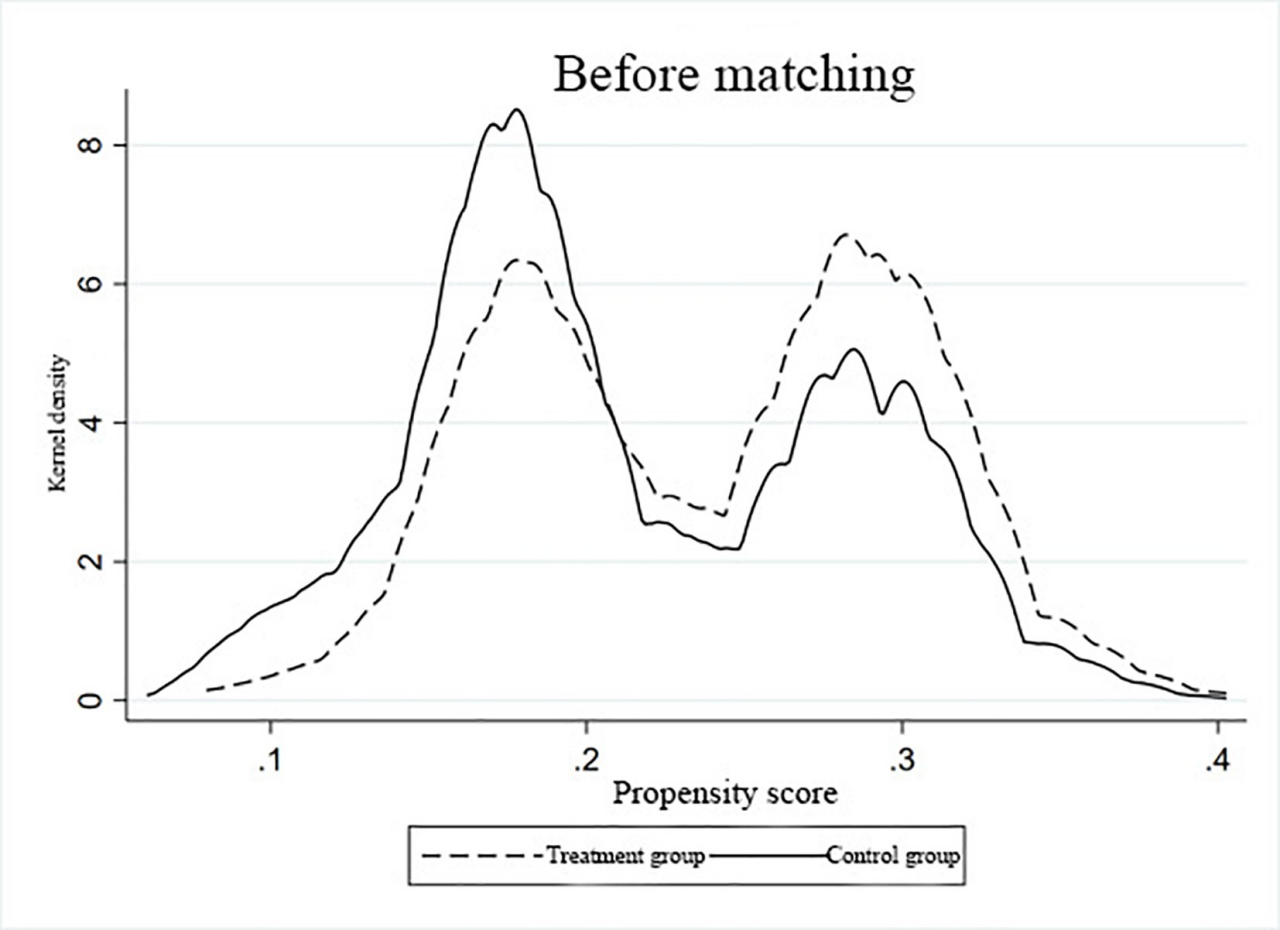
Befor-matching kernel density function graph.

**Fig 3:**
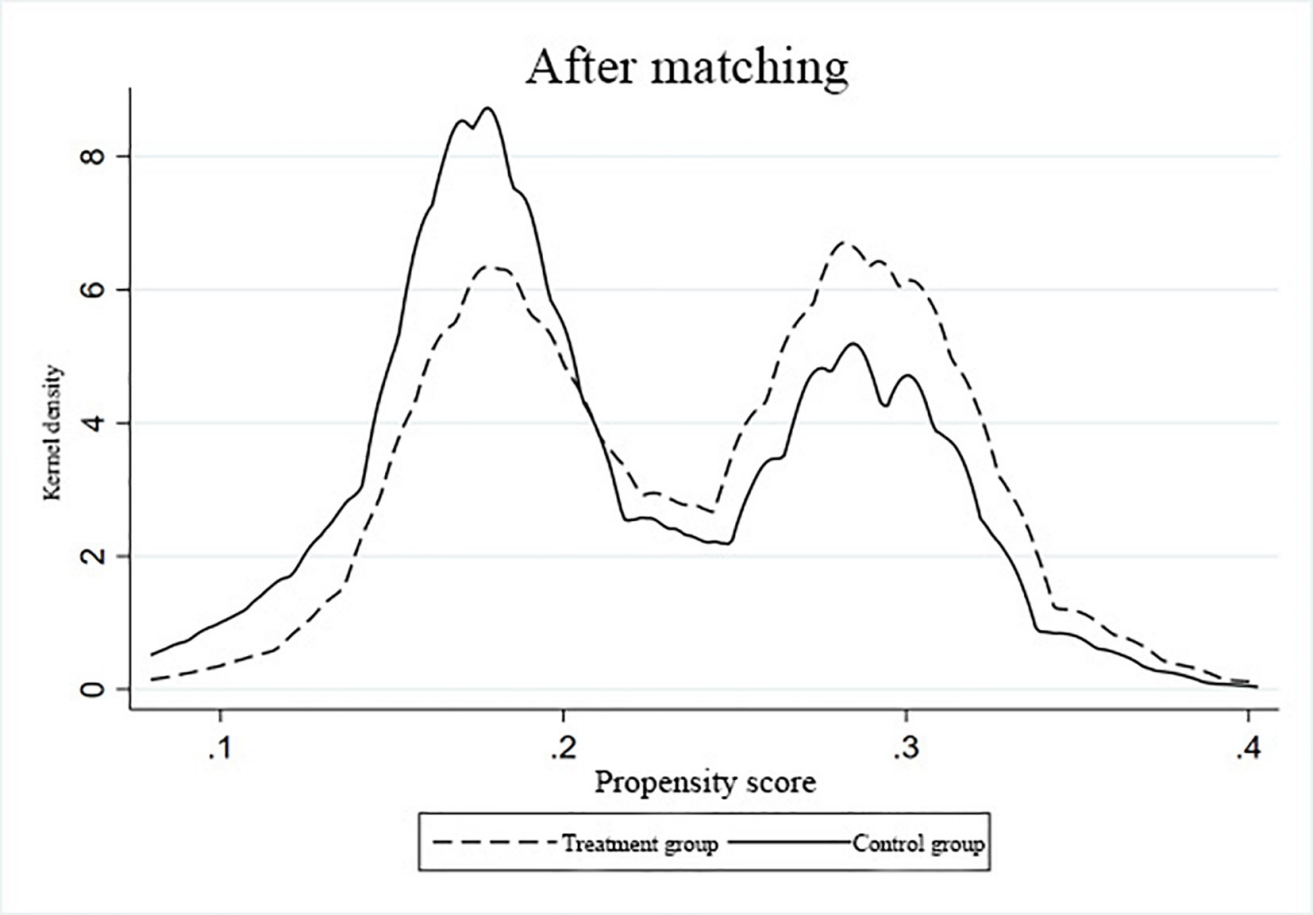
After-matching kernel density function graph.

Given that such structure did not determine differences in wealth distribution in families with pre-existing unequal distribution, the entire sample was matched. The common support domain test applied in this study is illustrated in Figs 2 and 3. The kernel density curves obtained after k-nearest neighbor matching displayed minimal loss of samples during propensity score matching, thereby satisfying the assumption of a common support domain.

**Fig 4:**
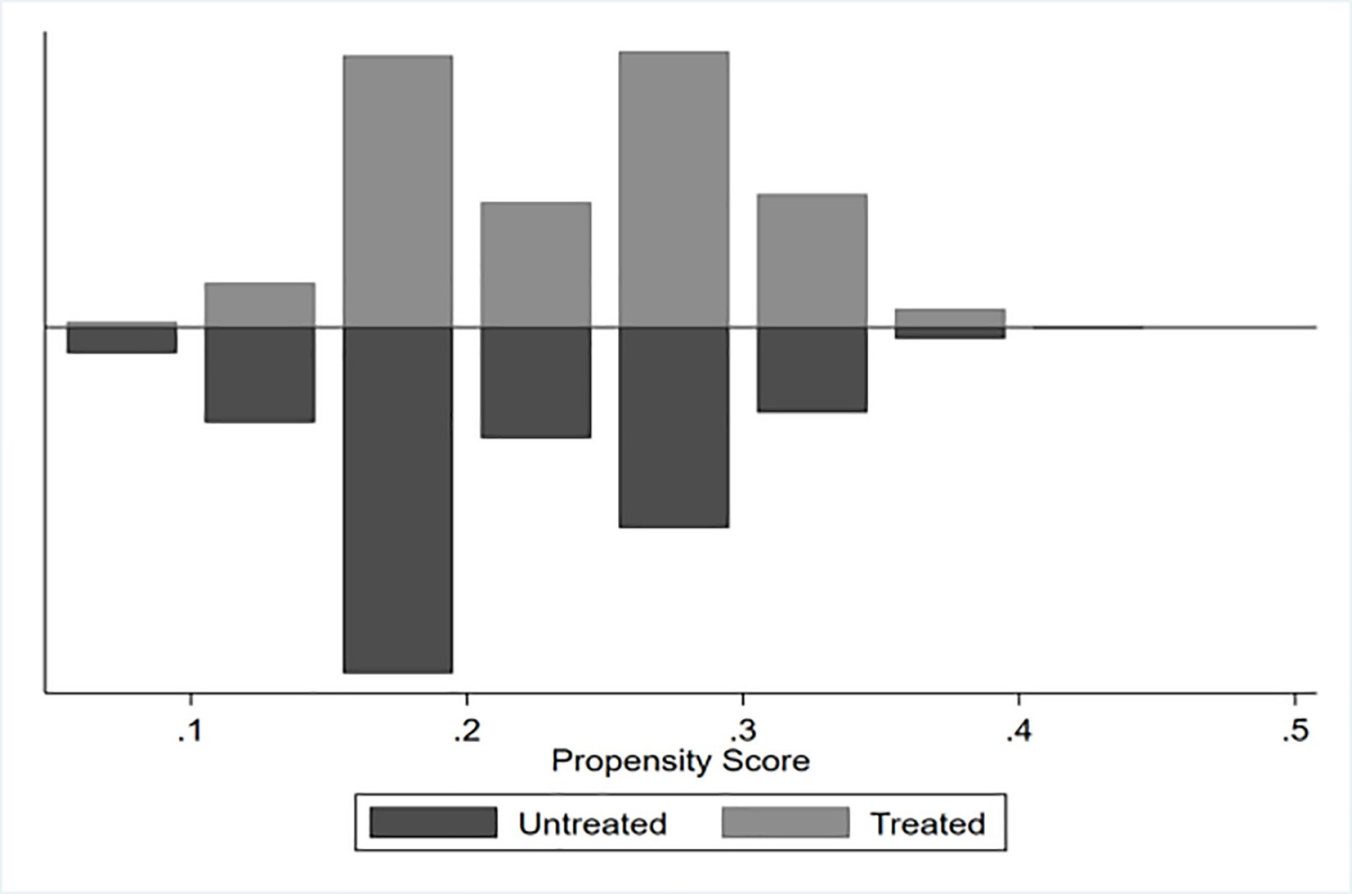
Common propensity score range.

The propensity score matching results are reported in Table 6, and the estimated results obtained using four different matching methods were highly consistent with the previously reported regression conclusions. Specifically, the average treatment effect indicated that the treatment group’s equal property distribution intention was 5.7–12.0 percentage points lower than that in the control group, and the absolute *t*-values all exceeded the critical value of 1.96, thus passing the significance test. The matching balance test results indicated that compared with pre-matching, the value of *Pseudo*–*R*^2^ decreased from 0.025 to 0.000; *LR chi*^2^ decreased from 283.02 to 1.02–3.15; the mean deviation decreased from 10.8 to 0.6–1.3, both of which were less than 10%; and the median deviation decreased from 10.4 to 0.3–0.8. The *B* values were less than 25%. Evidently, the bias caused by post-matching sample self-selection was significantly reduced, and no significant statistical difference was observed between the covariables. Data matching had a good balance. The common propensity score value range is presented in Fig 4. After matching, all samples were within the common value range, which further indicates the robustness of the matching effect.

**Table 6:** Propensity score matching.

Matching method	Treatment group VS Control group Daughters > Sons VS Daughters ≤ Sons		*ATT*	*S*.*E*	*T*−*stat*
1:1 match	0.381	0.438	-0.057	0.012	-4.84
K-nearest neighbor match(k = 4)	0.381	0.439	-0.058	0.012	-4.95
1:4 match (0.02 calliper)	0.381	0.501	-0.120	0.047	-2.53
kernel match	0.381	0.439	-0.058	0.012	-4.98
	Matching balance test				
	*Pseudo*−*R*^2^	*LR chi* ^2^	*MeanBias*	*MedBias*	*B* values
Before matching	0.025	283.02	10.8	10.4	40.1*
1:1 match	0.000	1.13	0.7	0.6	3.1
K-nearest neighbor match (k = 4)	0.000	1.02	0.6	0.3	2.9
1:4 match (0.02 calliper)	0.000	1.13	0.7	0.7	3.1
kernel match	0.000	3.15	1.3	0.8	5.2

### Heterogeneity analysis

The estimated outcomes of the heterogeneity analysis concerning the impacts of child structure and retirement mode on family property distribution intentions are presented in Table 7.

**Table 7:** Heterogeneity analysis results.

	Age (A)				Domicile (B)		Gender (C)	
	-30	31–40	41–50	51-	Village	City	Female	Male
Gender structure	0.636 (0.667)	-0.184 (0.167)	-0.270** (0.122)	-0.137* (0.081)	-0.175** (0.069)	-0.179 (0.131)	-0.173** (0.084)	-0.154* (0.088)
No. of children	-0.334 (0.329)	-0.020 (0.100)	-0.072 (0.069)	-0.106*** (0.037)	-0.070** (0.035)	-0.057 (0.060)	-0.019 (0.040)	-0.122*** (0.045)
Method of eldercare	-0.754** (0.319)	-0.222** (0.111)	-0.190** (0.095)	-0.290*** (0.079)	-0.288*** (0.056)	-0.314** (0.139)	-0.276*** (0.066)	-0.291*** (0.090)
Time effect	Control	Control	Control	Control	Control	Control	Control	Control
Regional effect	Control	Control	Control	Control	Control	Control	Control	Control
No. of samples	Control	Control	Control	Control	Control	Control	Control	Control
Gender structure	373	2502	3232	4561	8025	2664	5682	5004

The estimated results in Part A revealed differences in the effects of child structure and retirement mode on equal property distribution intentions at different ages. The influence of retirement mode on the intention to distribute family property equally maintained good robustness across different age groups, possibly due to the longstanding, solidified retirement arrangement in China. With aging, the demand for a family pension gradually increased, and the negative impact on the intention to distribute family property equally also gradually increased. Moreover, with aging, the influence of child structure on family property distribution intentions changed from positive to negative, with its significance initially increasing then decreasing. The number of children negatively affected equitable family property distribution intentions across all conditions, with its significance increasing with aging. Women commonly reproduce at the age of 30 years old or below, with child structure and the number of children not significant at that stage. The age range of 31–40 years old represents the stage of childbearing completion and child-rearing. Even though the child structure and the number of children were not significant during this time, the influence of the child structure changed from positive to negative. In the age range of 41–50 years, the child structure inhibited the equal distribution of family property at a 5% significance level, but the number of children had no significant effect. At age 51 years and above, which represents the stage of child-feeding and property redistribution, the child structure and number of children inhibited the equal distribution of property at the 10% and 1% significance levels, respectively.

The estimated results in Part B revealed no significant difference between agricultural and non-agricultural households regarding the influence of retirement mode on the intention to distribute family property equitably, with significance at the 1% and 5% levels, respectively. However, the child structure had a more significant inhibitory effect on agricultural households’ equitable property distribution intentions, both at the 5% level. No significant effect on non-agricultural households was determined, possibly due to cultural, economic, fertility, and other differences between urban and rural areas.

According to the estimation results in Part C, no significant gendered differences were documented regarding the influence of retirement mode on families’ equitable property distribution intentions, significant at the 1% level; however, the gendered difference in the influence of child structure on equitable family property distribution intentions was large. Among the factors, the negative effects on females and males were significant at the 5% and 10% levels, respectively. The number of children had a small, insignificant effect on women’s equitable property distribution intentions but significantly inhibited that for men, with significance at the 1% level. Therefore, women’s equitable property distribution intentions were greatly influenced by their children’s gender structure, whereas men’s were influenced by both the child structure and the number of children.

## Discussion

This study used data from the second and third phases of the China Women’s Social Status Survey to perform an in-depth analysis of the impacts of child structure and retirement mode on family property distribution intentions from both theoretical and empirical perspectives. After considering endogeneity issues and conducting robustness tests, we further explored the moderating mechanism of children’s surname selection and derived the following research conclusions.

Child structure, including children’s gender composition and the number of children in the family, significantly influenced family property distribution intentions. The child structure consistently inhibited equal wealth distribution in families, and this finding remained robust after addressing endogeneity concerns and conducting robustness tests. Our findings align with previous research, indicating that family wealth distribution tends to favor sons or that investment in sons is higher than in daughters [1, 30–32]. This may be because men typically have more influence over family decisions, and fathers, as elderly male figures, are more likely to be involved in decisions regarding the educational attainment of family members [33, 34]. Consequently, educational investments or family assets allocated to sons are often greater than those allocated to daughters.

Further, in families with pre-existing gender discrimination, the child structure did not significantly affect whether daughters received property, but the number of children significantly inhibited property allocation to daughters. This implies that in families with inequality, a higher number of children significantly reinforces the concentration of family wealth toward sons. This may be because, in families with a pre-existing preference for sons, boys are entrusted with the mission of carrying on the family lineage. As the number of children increases, parents tend to concentrate more of their resources on sons [32].

Retirement mode also profoundly affected family property distribution intentions. Opting for family-based eldercare significantly hindered equal wealth distribution within families. A higher number of children and proportion of female children also significantly inhibited equal wealth distribution. In families with pre-existing gender discrimination, choosing family-based eldercare intensified inequality, and having more children significantly decreased the possibility of daughters inheriting property. According to the literature, the likelihood of parents receiving care from their children in old age increased with the number of children [10, 17]. However, some studies argued the opposite, suggesting that as the number of children increased, each child’s financial and time support for the parents decreased due to the shirking of responsibility among siblings [9]. Notably, these studies only examined the impact of the number of children. In China, however, there might be a parental gender preference, with sons typically being assigned the responsibility of caring for elderly parents. Consequently, families choosing family-based elder care rely on sons, suppressing the equal distribution of family assets [35].

Regarding the moderating effects, as a measure of the changes in traditional cultural practices, surname selection partially weakened the impact of child structure on whether family wealth would be distributed equally. The results suggested that the decline in traditional beliefs has favored equal wealth distribution within families, but its effect on families with pre-existing discriminatory practices has not been significant. The results of this study align with existing research, demonstrating that as societal awareness of gender equality increased, the status of women gradually improved. This progress contributed to the equitable distribution of family assets among offspring, ensuring that girls and boys have equal probabilities of receiving family investments or inheritances [36].

Heterogeneity analysis revealed substantial variations in the impact of child structure on whether family wealth would be distributed equally. Agricultural households and those with family members aged 41 years old and above exhibited stronger inclinations toward unequal wealth distribution. Additionally, the gender disparity in family property distribution intentions was more pronounced in female-headed households. These findings align with existing research, which indicates that heterogeneity in family decision-making activities exists across different age groups, household registration types (rural/urban), and genders. This heterogeneity subsequently affects decisions regarding the allocation of family assets [33, 37, 38].

Existing studies predicted that the diminishing child structure differences, given the trend of declining family size, will impact the reallocation of family resources [39]. Therefore, challenging traditional gender norms, practicing gender equality from the ethical and cultural perspectives, and providing daughters with equal inheritance rights are necessary. Furthermore, with the rapidly aging population and the insufficient number of rural pension systems, the practice of relying on children for old age support remains prevalent. The concept of raising children for eldercare persists in families with both sons and daughters. This also contributes to the preference for sons over daughters. However, with the decline in the fertility rate, anticipated improvements in women’s economic status and caregiving abilities will likely narrow the gap in the provision of old age support between sons and daughters [40, 41]. This trend may alleviate gender discrimination in property distribution, thus emphasizing the importance of establishing a balanced relationship between rights and responsibilities.

Finally, we found that the decline in traditional beliefs encourages a more equitable distribution of family wealth, whereas the elevation of women’s status has yet to yield positive effects. While current practices still commonly involve assigning the paternal surname to children, growing gender equality is expected to empower women to compete for the right to use maternal surnames. To ensure equal inheritance rights for daughters, overcoming traditional gender norms, such as male succession, and eliminating the functional differences between sons and daughters are necessary. These changes contribute to building harmonious family relationships and play a crucial role in societal development.

Generally, altruism and exchange motivation are the two main motives guiding family property transfers [42, 43]. On one hand, the altruism model in family economics theory interprets parents’ contributions to their children as selfless and altruistic [44]. In traditional Chinese families, the main direction of economic flow within the family is from parents to children, and economic decisions are largely oriented toward children’s interests. On the other hand, owing to the expectation of reciprocity, parents may transfer property in exchange for filial support, and economic decisions are strongly influenced by retirement strategies [45, 46]. Some scholars have highlighted that altruistic property distribution intentions may be crowded out by the motivation of securing family support for seniors, whereas intentions motivated by exchange may increase due to the growing need for senior care. Family property distribution intentions are mostly influenced by these motivations [47].

### Strengths and limitations

We conducted empirical analyses incorporating the mechanisms underlying family property distribution intentions based on different child structures and number of children. We further explored the effect of child structure on family property distribution intentions amidst the evolving traditional views and the rise of women’s status, with surname choice and women’s status as moderating mechanisms. Furthermore, we found that relying on family members for old age support significantly influenced intergenerational wealth transfer. To the best of our knowledge, literature on the effect of eldercare on family property distribution intentions is limited. This study contributes to the field by exploring this relationship based on different child structures in families. Lastly, we investigated wealth distribution patterns in families with pre-existing gender discrimination. Heterogeneity analyses considered individual differences across various age groups, household types, and gender compositions; the conclusions remained valid after addressing endogeneity and conducting robustness checks.

The study has the following limitations. First, owing to data limitations, family property distribution intention was measured using a single indicator. Future studies should adopt a more solid, comprehensive approach. Second, this study only considered two moderating factors, children’s surname choice and women’s status; however, other factors, such as a rural versus an urban environment, may also play a moderating role. By including more variables, future research could improve the understanding of family property allocation intentions. Despite these limitations, our findings are valuable as they clearly demonstrate that child structure and means of eldercare play decisive roles in family property distribution intentions.

## Conclusion

This study aims to investigate the distribution of family assets, correlating the structure of offspring and methods of eldercare with the intent to distribute family assets. To this end, a comprehensive analysis was performed across various age groups, household registration types, and genders, providing empirical evidence and data to support equitable inheritance between sons and daughters.

Comparative studies across various countries were conducted. Accordingly, the study finds that the preference for boys in China leads to an inequitable distribution of family assets. This conclusion aligns with existing research that has observed a preference for sons in developing countries. However, such gender preferences were not observed in developed countries. Furthermore, the present study observes that an increase in the number of children can mitigate the concentration of family assets driven by gender preferences. Notably, comparative studies between developed and developing countries reveal consistent findings. For example, research by Raley and Bianchi [30] using a US sample found that in the United States, as the number of daughters increased, fathers’ investments in their sons diminished over time.

It is worth noting that prior studies have often failed to adequately consider the potential average differences between sons and daughters in both study design and the interpretation of results, potentially leading to differential parental treatment. For future studies, greater emphasis on how sons and daughters elicit or amplify differences in parental investment is a topic that deserves more rigorous sociological investigation.

Finally, this study recommends that in developing countries like China, necessary policies and interventions be designed to establish ethical and cultural equality between girls and boys. Equally important, girls should be provided equal inheritance rights, and children’s duty to support their parents should be aligned with their right to inherit property.
